# Test-retest reliability of modular-relevant analysis in brain functional network

**DOI:** 10.3389/fnins.2022.1000863

**Published:** 2022-12-07

**Authors:** Xuyun Wen, Mengting Yang, Liming Hsu, Daoqiang Zhang

**Affiliations:** ^1^College of Computer Science and Technology, Nanjing University of Aeronautics and Astronautics, Nanjing, Jiangsu, China; ^2^MIIT Key Laboratory of Pattern Analysis and Machine Intelligence, Nanjing, Jiangsu, China; ^3^Center for Animal MRI, University of North Carolina, Chapel Hill, Chapel Hill, NC, United States

**Keywords:** test-retest reliability, brain functional network, module detection, modular structure, network metric

## Abstract

**Introduction:**

The human brain could be modeled as a complex network *via* functional magnetic resonance imaging (fMRI), and the architecture of these brain functional networks can be studied from multiple spatial scales with different graph theory tools. Detecting modules is an important mesoscale network measuring approach that has provided crucial insights for uncovering how brain organizes itself among different functional subsystems. Despite its successful application in a wide range of brain network studies, the lack of comprehensive reliability assessment prevents its potential extension to clinical trials.

**Methods:**

To fill this gap, this paper, using resting-state test-retest fMRI data, systematically explored the reliabilities of five popular network metrics derived from modular structure. Considering the repeatability of network partition depends heavily on network size and module detection algorithm, we constructed three types of brain functional networks for each subject by using a set of coarse-to-fine brain atlases and adopted four methods for single-subject module detection and twelve methods for group-level module detection.

**Results:**

The results reported moderate-to-good reliability in modularity, intra- and inter-modular functional connectivities, within-modular degree and participation coefficient at both individual and group levels, indicating modular-relevant network metrics can provide robust evaluation results. Further analysis identified the significant influence of module detection algorithm and node definition approach on reliabilities of network partitions and its derived network analysis results.

**Discussion:**

This paper provides important guidance for choosing reliable modular-relevant network metrics and analysis strategies in future studies.

## Introduction

The human brain could be modeled as a complex network *via* functional magnetic resonance imaging (fMRI) consisting of brain regions as nodes linked by edges, estimated with functional connectivity (FC) (Friston, 2011; Park and Friston, 2013). As with other real-world connected systems, investigating the topology of brain functional interactions has profound implications for understanding its complex principles (Heuvel and Pol, 2010), e.g., underlying neural-mechanisms of human complex behaviors and cognitive capacities (Bullmore and Sporns, 2009) and pathogenesis of brain diseases (Sang et al., 2015; Dai et al., 2018), etc. The brain functional network can be studied from different spatial scales with graph theory tools, such as global network efficiency at macroscale (van den Heuvel et al., 2009), functional sub-systems’ coordination at mesoscale (Chan et al., 2014), and regional properties at microscale (Cheng et al., 2015). In recent decades, network metrics at each scale have provided crucial insights for understanding the organization of human brain in health and disease.

With the extension of the use of FCs coupled to graph theory analysis to clinical trials becoming possible, increasing attention has been given to the reliability of these studies (Braun et al., 2012; Liang et al., 2012; Liao et al., 2013; Cao et al., 2014; Andellini et al., 2015; Du et al., 2015; Wang et al., 2017; Jin et al., 2018; Zeng et al., 2019). Test-retest reliability (TRT), which quantifies within-subject stability of a measure under multiple occasions in a group of subjects (Lavrakas, 2008), has been widely used for reliability assessment of fMRI data analysis. Based on this approach, the robustness of various graph-theory analyses at different spatial scales in functional brain networks has been extensively explored over the past decade. Macroscale studies found that most global network metrics, including global efficiency, clustering coefficient, characteristic path length, small-worldness, modularity, and hierarchy, are reproducible with reliability ranging from moderate to good (Braun et al., 2012). For nodal topological properties at microscale, their reliabilities displayed a spatially heterogeneous distribution, wherein regions in association areas exhibit moderate reliability (Liang et al., 2012), and nodal degree and nodal efficiency are more reliable than nodal betweenness (Liao et al., 2013). Compared to macroscale and microscale network metrics with their TRT reliability systematically assessed, network properties at mesoscale still lack a comprehensive reliability evaluation.

In recent years, module detection is becoming a popular approach for investigating mesoscale properties of brain functional networks (Gallos et al., 2012; Park and Friston, 2013; Sporns and Betzel, 2016; Baum et al., 2017). It decomposes the human brain into several functional modules, each of which represents groups of brain regions that perform specialized neurophysiological function (Power et al., 2011; Cole et al., 2014). Following suits, a variety of module-related network metrics were proposed, including modularity (Sporns and Betzel, 2016), intra- and inter-modular FCs (Ma et al., 2017), within-modular degree, and participation coefficient (Guimera and Amaral, 2005). More importantly, these measurements have been used as biomarkers for brain disease diagnosis. For example, previous studies reported abnormal changes of modularity, intra- and inter-modular FCs in neurological ailments like Alzheimer’s disease (Haan et al., 2012), Parkinson’s disease (Cai et al., 2018), and depression (Andric and Hasson, 2015). Furthermore, the detected modular structure also influences the classification of nodes into different functional roles through within-modular degree (reflecting intra-modular coordination) and participation coefficient (reflecting inter-modular coordination) (Meunier et al., 2009a; Bertolero et al., 2015; Wen et al., 2019). These hub regions have been found to have increased susceptibility in case of brain disorders and cause major functional disruption in case of brain injury (Power et al., 2013). All abovementioned studies suggest the promising future of modular-relevant network analysis, and timely assessment of its reliability is crucial for its broader applications because only with adequate reliability, we can then expect the detected modular-relevant biomarkers to be reproducible.

The reliability of modular-relevant analysis in brain functional networks depends on two factors, i.e., the reliability of edges (i.e., FCs among brain regions) and the repeatability of network partition. TRT reliability regarding to FCs have been well assessed, which can be affected by many factors, including imaging acquisition parameters (Shirer et al., 2015), imaging preprocessing steps (Parkes et al., 2017), and FC estimation methods (Liang et al., 2012; Fiecas et al., 2013; Mejia et al., 2018), etc. A recent review literature (Noble et al., 2019) concludes that fMRI studies with high reliability tend to have the following characteristics: (1) eyes open and awake recordings of subjects during imaging scanning, (2) as much frames of fMRI data for each subject as possible, (3) test and retest measurements collected at shorter inter-scan intervals, and (4) connectivity estimated *via* full correlation. This study provides an important guidance for choosing the effective approach to improve the reliability of FCs. In contrast to systematic studies of FC reliability, how to guarantee the repeatability of network partition in brain functional networks remains unclear. It is well known that, the non-deterministic nature of module detection is an NP-hard problem (Xu et al., 2010; Gong et al., 2014), the reliability and quality of its resultant network partition is potentially affected *module searching strategy* and *network size*. Therefore, in reliability evaluation of modular-relevant analysis, the influence of module detection algorithm and node definition approach should be highlighted.

In this work, we systematically explored the reliability of various modular-relevant network measurements, including modular structure and five modular-relevant network properties. We aimed to investigate the following three questions: (1) *Are individual- and group-level modular-relevant network analysis methods reliable and reproducible*? (2) *Does the module detection algorithm affect reliability of modular-relevant measurements*? (3) *Does the node definition approach affect reliability of modular-relevant measurements*? To this end, we used a test-retest fMRI dataset from 45 healthy subjects in Human Connectome Project (HCP) database and adopted three coarse-to-fine brain atlases to construct individual brain functional networks. For each kind of networks, we used four individual-level module detection methods and 12 group-level module detection methods to capture the corresponding network partitions and then calculated the related network measurements. TRT reliability was computed with intra-class correlation coefficient (ICC).

## Materials and methods

**FIGURE 1:**
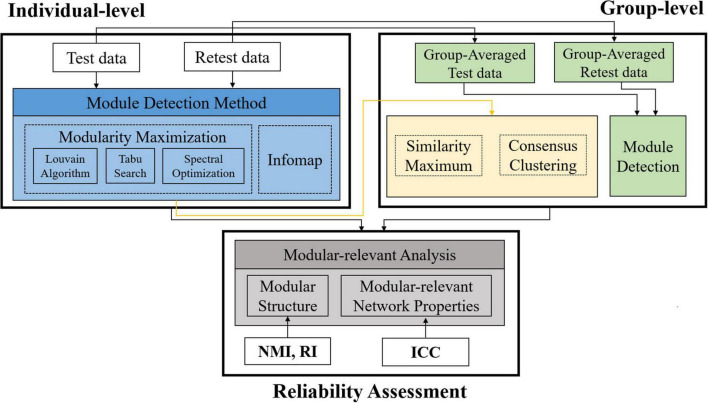
Summary of the processing pipelines in this paper. First, we constructed three types of brain functional networks for each subject based on different brain atlases. Second, we detected modular structure for each subject with four methods and for the population group with 12 methods (i.e., three group-wise modular construction frameworks four module searching approaches). Third, we computed five modular-relevant network properties, including modularity, intra- and inter-modular functional connectivities (FCs), within-modular degree, and participation coefficient. Finally, we assessed the test-retest and reliability of each kind of estimations with different measurements. NMI, normalized mutual information; RI, rand index; ICC, intra-class coefficient.

A summary of the processing pipelines in this paper was given in Figure 1. Specifically, based on a publicly available HCP test-retest database, we first conducted module detection on each single subject and the population group to obtain individual- and group-level modular structures, and then calculated five popular modular-relevant network properties. After that, we assessed the reproducibility of all above estimations to give a comprehensive evaluation of TRT reliability of modular-relevant analysis methods in brain functional networks.

### Participants

This study was carried out using test-retest dataset from the publicly available HCP database S1200 release^1^ (Van Essen et al., 2013). It includes 45 healthy subjects (13 males, aged 22∼35 years old) with full 3T imaging scans. All participants were free of current psychiatric or neurologic illness. Extensive descriptions of the data, please refer to Marcus et al. (2013).

Each subject has two resting-state fMRI (rs-fMRI) sessions, each of which consists of two runs scanned with the same multi-band sequence (Moeller et al., 2010) but the different readout directions (i.e., one with LR phase coding direction and the other with RL direction). The mean interval between two sessions of subjects is ∼140 days. The first section is used as test data and the second session is used as retest data. All rs-fMRI data were collected with eye open and relaxed fixation on a projected bright cross-hair on a dark background. The other acquisition parameters for rs-fMRI were: TR = 720 ms, TE = 33.1 ms, flip angle = 52°, voxel size = 2 mm^3^ (isotropic), 72 slices, and total volumes = 1,200 (15 min). For more detailed information of rs-fMRI in HCP, please refer to the previous work (Smith et al., 2013).

### Data preprocessing

All rs-fMRI data were first preprocessed by “HCP fMRIvolume” minimal preprocessing pipeline (Glasser et al., 2013), including the following procedures: (1) gradient distortion correction, (2) head motion correction, (3) EPI distortion correction, (4) registration to the Montreal Neurological Institute (MNI) space, (5) intensity normalization to a global mean, and (6) masking out non-brain voxels. After that, we further adopted independent component analysis (ICA) based FIX Xnoiseifier to remove artifacts from fMRI data (Griffanti et al., 2014; Salimi-Khorshidi et al., 2014). During this cleanup, 24 head motion parameters (including 6 rigid -body motion parameters, their backward temporal derivatives, and squares of those 12 time series) and “bad components” estimated from ICA were regressed from blood oxygen level dependent (BOLD) signals of each scan.

### Brain functional network construction

Considering the node definition approach may affect repeatability of module detection methods, this paper adopted three different types of brain atlases to define nodes in brain functional networks. These three atlases were defined with different methods (or brain features) and parcellate the human brain from a coarse to fine scale. They are (1) *automated anatomical labeling (AAL)* atlas derived from anatomical landmarks and parcellate the human brain into 90 brain regions (Tzourio-Mazoyer et al., 2002); (2) *Shen’s FC-based atlas* generated from rs-fMRI data and includes 217 brain regions (Shen et al., 2013); (3) *Zalesky’s random atlas* defined with the random parcellation method and consists of 1,024 brain regions (Zalesky et al., 2010). Notably, we excluded cerebellar regions of AAL atlas and Shen atlas due to the difficulty of cerebellum registration.

After obtaining parcels with brain atlas, we constructed the corresponding functional brain network for each scan using the following steps. Specifically, we first extracted rs-fMRI time series for each brain region (i.e., brain parcel) by averaging blood oxygen level dependent (BOLD) signals of all voxels within it. We then computed the Pearson’s correlation between each pair of time series and used them as edges of brain functional networks. After that, we conducted *r*-to-*z* value conversion on each FC matrix with Fisher-z transformation to improve the data normality. Since each session has two runs, each subject has two FC matrices, one of which was generated from rs-fMRI data with LR readout direction and the other from RL direction. We followed the suggestion in Smith et al. (2013) to construct the representative FC network for each subject by averaging LR and RL FC matrices. Finally, for each subject, we set all the negative values of FC network of each subject to zeros for their unclear biological meanings (Garrison et al., 2015). In summary, each subject in each dataset would have a 90 ×90 FC matrix, a 217 ×217 FC matrix, a 1,024 ×1,024 FC matrix. It should be noted that the weighted brain networks were used in the following experiments.

### Module detection algorithms

The module detection in brain networks is broadly divided into two categories, one of which is to detect modular structure for one single subject (i.e., individual-level module detection) and the other is to generate a common network partition for a cohort of subjects (i.e., group-level module detection). For a comprehensive evaluation, we assessed TRT reliability of modular-relevant analysis at both individual and group levels. For each category, we selected a variety of widely used module detection methods to generate different network partition results to evaluate the influence of module detection algorithm on reliability. To ensure the quality of network partitions, we only kept top 10% strongest positive connections to ensure the sparsity of FC networks (Wen et al., 2019; Zhang et al., 2021). After obtaining individual- and group-level modular structures, we projected them on brain surface for further comparison between different methods and different brain atlases. The brain parcellation template generated on a large population by using the clustering method was adopted as ground truth (Yeo et al., 2014), whose spatial distribution is displayed in Supplementary Figure 1.

#### Individual-level module detection algorithms

Among various module detection algorithms, “modularity maximization” is the most widely adopted method in brain module detection in FC-related studies (Sporns and Betzel, 2016). This approach divides the network into modules by maximizing modularity quality function, during which the optimization strategy has an effect on the quality of the solution. Considering this situation, this study employed three different approaches to search for optimal modularity, including Louvain algorithm (Blondel et al., 2008), Tabu search (Arenas et al., 2008), and spectral optimization (Newman, 2006). For each approach, we repeated module detection 50 times on each network, and used consensus clustering to construct consistent modules to avoid their uncertainty (Bassett et al., 2013). In addition to the above three methods, we also used Infomap algorithm for module detection due to its good performance in other domains (Rosvall and Bergstrom, 2008). Unlike the first three methods, Infomap detects modules by minimizing the description length of random walks in the network. In summary, for each single subject, we employed four module detection methods to capture its modular structure.

#### Group-level module detection algorithms

Currently, there are three popular group-wise frameworks for determining modular structure on the population group, which can be named as “Ave,” “SimMax,” and “Consensus,” respectively. “Ave” based methods first construct a representative brain network by averaging FC matrixes across all subjects, and then apply module detection on this averaged network for generating group-level modular structure (Meunier et al., 2009b). “SimMax” based methods conduct module detections on all subjects and choose the partition that is most similar to others as the group-level result (Meunier et al., 2009b). “Consensus” based methods first perform module detection for each subject to construct the association matrix, where each element represents the probability of assigning a pair of nodes to the same module across subjects, and then employ the second clustering on association matrix to generate group-level network partition (Bassett et al., 2013). By combining three group-wise modular construction schemes with four individual-level module detection methods, there are 12 group-level module detection methods, named as Louvain + Ave, Tabu + Ave, Spectral + Ave, Infomap + Ave, Louvain + SimMax, Tabu + SimMax, Spectral + SimMax, Infomap + SimMax, Louvain + Consensus, Tabu + Consensus, Spectral + Consensus, Infomap + Consensus.

### Modular-relevant network properties

Based on the detected module structure, we computed five popular network properties, including modularity, intra-modular FC, inter-modular FC, within-modular degree, and participation coefficient. Among these metrics, the first three assessed the information coordination capacity among functional modules of the whole brain network (including separation and integration), and the latter two evaluated the topological role of nodes within module and between different modules, which are regional level measurements. There are differences for network property calculation between individual and group levels. Specifically, for a given network, individual-level network metrics were computed based on modular structure detected on itself, while group-level network metrics were measured by warping group-wise representative on the network. All positive connections in FC network were used for computing network properties.

#### Modularity

It is defined as the difference between the fraction of edges within the given modules and the expected fraction if edges are distributed at random (Newman, 2006), which is formulated as


(1) $$Q=\frac{1}{2\,m}\,\sum_{\mathrm{ij}}\left(a_{\mathrm{ij}}-\frac{k_{i}\,k_{j}}{2\,m}\right)\,\delta \,\left(\sigma_{i},\sigma_{j}\right)$$

where *a*_*ij*_ represents the link strength between nodes *i* and *j*, and *k*_*i*_ indicates the degree of node *i*. *m* is the total link strength of the whole network. The Kronecker delta function, δ(σ_i_,σ_j_), is equal to 1 when nodes *i* and *j* belong to the same module; otherwise, it is set to 0. Modularity is a network-level measurement for evaluating network segregation.

#### Intra-modular functional connectivity

It measures the total link strength of FC within the modules, which is defined as the averaged FC strength across all the within-modular connections (Chan et al., 2014). This index is generally used for evaluate network segregation.

#### Inter-modular functional connectivity

It measures the total link strength of FC between different modules, which is defined as the averaged FC strength across all the edges that linked different modules (Chan et al., 2014). This index can be used as an indicator for network integration.

#### Within-modular degree

It characterizes the importance of a region within its own module (Guimera and Amaral, 2005). Given a modular partition 𝒞 = {*c*_1_, *c*_2_,…,*c*_*A*_}, the within-module degree *WD* of a node *n* in module *c*_*i*_ is formulated as


(2) $$W\,D_{n}=\frac{K_{c_{i}}^{n}-\bar{K_{c_{i}}}}{\delta_{K_{c_{i}}}}$$

where ${\text{K}}_{{\text{c}}_{\text{i}}}^{\text{n}}$ is the total FC strength between node *n* and the other nodes in module *c*_*i*_, and $\bar{{\text{K}}_{{\text{c}}_{\text{i}}}}$ and δ_K_c_i___ represent the average and standard deviation of ${\text{K}}_{{\text{c}}_{\text{i}}}^{\text{n}}$ across all nodes in module *c*_*i*_, respectively.

#### Participation coefficient

It evaluates the region’s importance in connecting with different modules (Guimera and Amaral, 2005). Given a modular partition 𝒞 = {*c*_1_, *c*_2_,…,*c*_*A*_}, the participation coefficient of a node *n* in module *c*_*i*_ is defined as


(3) $$P\,C_{n}=1-\sum_{i=1}^{A}{\left(\frac{K_{c_{i}}^{n}}{k_{n}}\right)}^{2}$$

where ${\text{K}}_{{\text{c}}_{\text{i}}}^{\text{n}}$ is the total FC strength between node *n* and the other nodes in module *c*_*i*_ and *k*_*n*_ is the sum of FC strength connecting node *n*.

### Reliability assessment

To systematically assess the reliability of modular-relevant analysis in brain functional networks, we computed the reproducibility of three network analysis methods by using different evaluation indexes, including modular structure and modular-relevant network metrics.

#### Modular structure

The repeatability of modular structure between test and retest data was evaluated with normalized mutual information (NMI, Ana and Jain, 2003) and rand index (RI, Rand, 1971). Suppose there are two partitions 𝒜 = {*c*_1_, *c*_2_,…,*c*_*A*_} and ℬ = {*c*_1_, *c*_2_,…,*c*_*B*_}. NMI is an information-theory-based index, which is calculated as follows:


(4) $$N\,M\,I\,\left(𝒜,ℬ\right)=\frac{-2\,\sum_{i=1}^{A}\sum_{j=1}^{B}N_{i\,j}\,l\,o\,g\,\left(\frac{N_{i\,j}\,N}{N_{i.}\,N_{.j}}\right)}{\sum_{i=1}^{A}N_{i.}\,l\,o\,g\,\left(\frac{N_{i.}}{N}\right)+\sum_{j=1}^{B}N_{.j}\,l\,o\,g\,\left(\frac{N_{.j}}{N}\right)}$$

In Eq. 4, ***N*** is confusion matrix, where each element *N*_*ij*_ denotes the number of nodes in module *c*_*i*_ of partition 𝒜 that appears in module *c*_*j*_ of partition ℬ. N_i._ is the sum over row *i* of matrix ***N*** and N_.j_ is the sum over column *j*. *N* is the sum over all elements in ***N***. Different with NMI, RI calculates the fraction of correctly classified elements to all elements, which can be formatted as


(5) $$R\,I\,\left(𝒜,ℬ\right)=\frac{2\,\left(n_{11}+n_{00}\right)}{n\,\left(n-1\right)}$$

where *n*_11_ is the number of node pairs that are in the same mode under 𝒜 and ℬ, and *n*_00_ is the number of node pairs that in different modules under 𝒜 and ℬ. *n* is the number of nodes in FC networks. Both NMI and RI scores range from 0 to 1, where 1 corresponds to identical network partitions between 𝒜 and ℬ, and 0 indicates two entirely dissimilar network partitions.

#### Modular-relevant network properties

The TRT reliability of five modular-relevant network properties were evaluated using two-way mixed single-measures intra-class correlation coefficient (ICC) (Shrout and Fleiss, 1979), which is defined as


(6) $$\mathrm{ICC}=\frac{\text{BMS}-\text{EMS}}{\text{BMS}+\left(\text{s}-1\right)\,\text{EMS}}$$

where *BMS* is the between-subjects mean square, *EMS* notates the error mean square and *s* is the number of repeated measurement (here, *s* = 2). ICC coefficient conceptually ranges from 0 (not reliable at all) to 1 (perfectly consistent between repeated measurements), but its estimation can be negative in a few cases. In this paper, we followed previous literatures and set the negative ICC values to be zeros (Zhang et al., 2011, 2017). Based on the values of ICC, reliability is usually categorized as poor (0∼0.2), fair (0.2∼0.4), moderate (0.4∼0.6), good (0.6∼0.8), and excellent (>0.8) (Koch, 1977; Chen et al., 2015).

## Results

### Reliability of individual-level modular-relevant analysis

**FIGURE 2:**
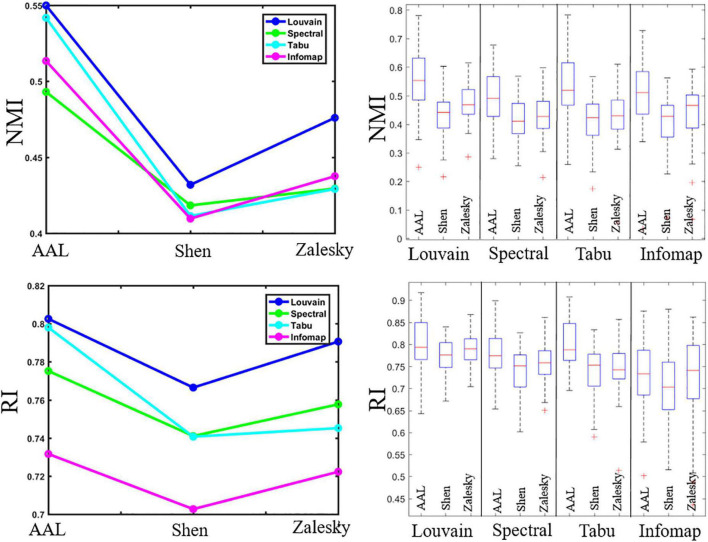
Repeatability of individual-level module detection results. **(Left)** Averaged reliability values obtained using normalized mutual information (NMI, **top**) and Rand index (RI, **bottom**). **(Right)** Box plots indicated the reliability across subjects on automated anatomical labeling (AAL), Shen and Zalesky atlases, from left to right for each method.

**FIGURE 3:**
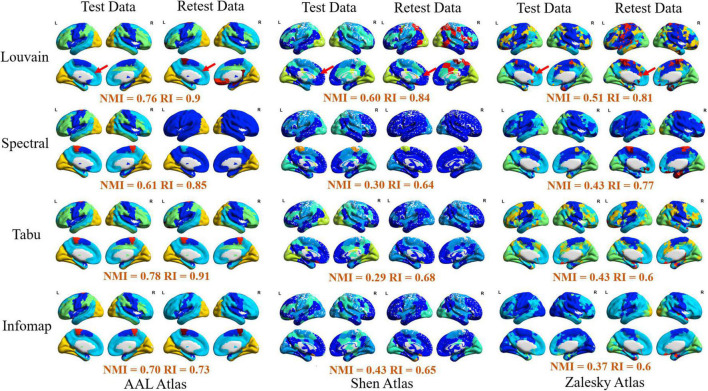
Visualization of modular structure detected by different methods on an exemplar subject (subject ID: 114823). In each sub-figure, different colors represented different functional modules, and the similarity of module detection results between test and retest data estimated by normalized mutual information (NMI) and rand index (RI) were given below the figure.

**FIGURE 4:**
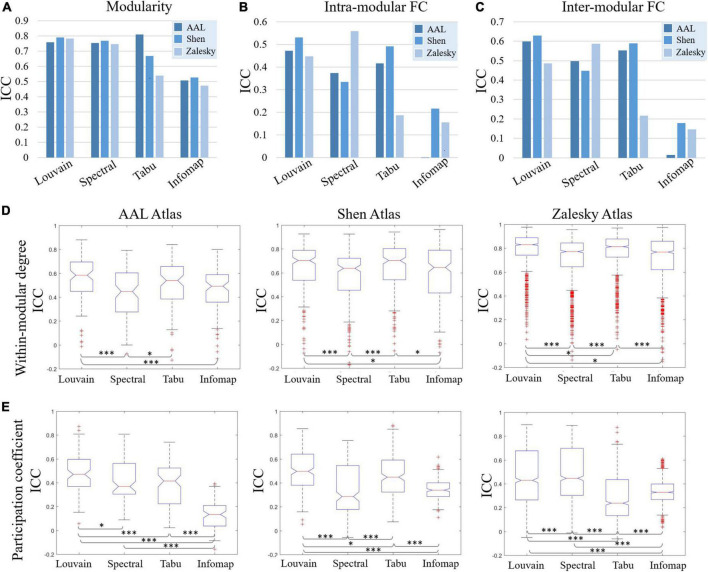
Test-retest reliability results of individual-level modular-relevant network metrics estimated by intra-class correlation (ICC), including **(A)** modularity, **(B)** intra-modular functional connectivity (FC), **(C)** inter-modular FC, **(D)** within-modular degree, and **(E)** participation coefficient. In panels **(D,E)**, two-sample *t-*test are used for method comparison, where * represents significant level *p* < 0.05 and *** represent *p* < 0.005.

In this section, we reported TRT reliability results of modular-relevant analysis on individual level, including modular structure and five modular-relevant network properties. Modular structure results were presented in Figures 2, 3. Modular-relevant network metric results, including modularity, intra- and inter-modular FCs, within-module degree and participation coefficient, were displayed in Figure 4 and Table 1.

**TABLE 1:** Rankings of four individual-level module detection algorithms on five network metrics estimated on three brain atlases.

Metrics	Atlas	ICC rank
Modularity	AAL	Tabu (excellent) > Louvain (good) > Spectral (good) > Infomap (moderate)
	Shen	Louvain (good) > Spectral (good) > Tabu (good) > Infomap (moderate)
	Zalesky	Louvain (good) > Spectral (good) > Tabu (moderate) > Infomap (moderate)
Intra-modular FC	AAL	Louvain (moderate) > Tabu (moderate) > Spectral (fair) > Infomap (poor)
	Shen	Louvain (moderate) > Tabu (moderate) > Spectral (fair) > Infomap (poor)
	Zalesky	Spectral (moderate) > Louvain (moderate) > Tabu (fair) > Infomap (poor)
Inter-modular FC	AAL	Louvain (moderate) > Tabu (moderate) > Spectral (moderate) > Infomap (poor)
	Shen	Louvain (good) > Tabu (moderate) > Spectral (moderate) > Infomap (poor)
	Zalesky	Spectral (moderate) > Louvain (moderate) > Tabu (poor) > Infomap (poor)
Within-module degree	AAL	Louvain (moderate) > Tabu (moderate) > Infomap (moderate) > Spectral (moderate)
	Shen	Louvain (good) > Tabu (good) > Spectral (good) > Infomap (good)
	Zalesky	Louvain (excellent) > Tabu (excellent) > Spectral (good) > Infomap (good)
Participation coefficient	AAL	Louvain (moderate) > Tabu (moderate) > Spectral (fair) > Infomap (poor)
	Shen	Louvain (moderate) > Tabu (moderate) > Infomap (fair) > Spectral (moderate)
	Zalesky	Spectral (moderate) > Louvain (moderate) > Infomap (fair) > Tabu (fair)

Poor reliability (0 < ICC < 0.2), fair reliability (0.2 < ICC < 0.4), moderate reliability (0.4 < ICC < 0.6), good (0.6 < ICC < 0.8), and excellent reliability (ICC > 0.8).

#### Modular structure

Figure 2 displayed the repeatability of individual-level module detection results computed by NMI and RI. For ease of comparison, we reported averaged evaluation measures in the form of line graphs for all computed results, and also showed box plots alongside line graphs to represent the variability across subjects.

In Figure 2, we observed that the repeatability of network partition results depends heavily on module detection algorithms. We could draw two conclusion. *First*, modularity maximization based methods yielded higher reliability than Infomap algorithm in almost all experiments, suggesting it is more suitable for module detection in brain functional network. *Second*, of three estimated modularity optimization strategies, Louvain algorithm displayed the highest reliability in all experiments, and its advantage became more and more prominent as the network size increases. Specifically, on the brain network with 90 nodes (i.e., AAL atlas), the Louvain algorithm performed slightly better than Tabu method, but with the number of nodes increases to 217 (i.e., Shen atlas), their gap became larger. When the number of nodes increases to 1,024 (i.e., Zalesky atlas), the performance of Louvain algorithm was significantly better than the other methods. This result implies that the choice of module detection algorithm is important to ensure the reliability of module detection results, especially for the large-scale network.

Regarding the brain atlas, we found that AAL atlas yielded the highest reliability in all experiments. This result is due to its much smaller number of nodes than the other two atlases reduces the difficulty of module detection. A surprising finding is that, in most of cases, Shen atlas with fewer nodes generated lower NMIs and RIs than Zalesky atlas with more nodes. This finding suggests that the brain atlas construction approach can also affect the repeatability of module detection on brain functional networks.

Furthermore, to verify the availability of module detection results by different methods on different brain atlases, we randomly selected an exemplar subject (subject ID: 114823) and visualized his/her module detection results on brain surface in Figure 3. We observed that (1) most of module detection results captured four available functional subsystems, including visual network (VN), sensorimotor network (SMN), default mode network (DMN), and frontal-parietal network (FPN), and (2) brain regions in each module derived from AAL atlas were more spatially contiguous than those from Shen and Zalesky atlases. Compared with Yeo’s atlas (Yeo et al., 2014), the modular structures detected on Shen and Zalesky atlases were closer to the true spatial distribution of functional systems than the AAL atlas. As red line labeled, AAL atlas incorrectly assigned a large brain region in frontal areas in SMN to DMN.

#### Modular-relevant network properties

Test-retest reliability on five network properties derived from individual-level module detection were given in Figure 4, where the results of modularity, intra- and inter-modular FCs were visualized as bar figures and the results of within-modular degree and participation coefficient were displayed as box plots. For ease of comparison, for each network metric estimated on each brain atlas, we ranked four individual-level module detection methods based on their ICC values as summarized in Table 1.

As shown in Figure 4 and Table 1, among five estimated network properties, modularity yielded the highest ICC values with the reliability above good level on all three brain atlases, and the reliability of the other four properties ranged from moderate to good. But overall, these network metrics derived from individual-level module detection in brain functional networks are reliable and reproducible.

We also observed a significant impact of module detection algorithm on the reliability of modular-relevant network metrics. Consistent with the repeatability of network partitions, Louvain algorithm performed best in reliability assessment experiments, and Infomap algorithm performed the worst. Specifically, as shown in Table 1, Louvain algorithm ranked first in 11/15 cases, whereas Infomap algorithm ranked last in 12/15 cases. For the other two methods, Tabu method yielded higher ICC values than spectral method on smaller networks (i.e., AAL atlas and Shen atlas), but lower ICC values on larger networks (i.e., Zalesky atlas), indicating it cannot handle the large-scale brain network.

Regarding to the influence of brain atlas on network metrics, a surprising finding is that, the reliability of modular-relevant network metrics was not significantly changed with the increase of network size, except within-modular degree. Take results from Louvian algorithm as the example for illustration. As shown in Figure 4, although ICC values of modularity, intra- and inter-modular FCs, and participation coefficient displayed variation across different brain atlases, these changes were not very significant, and their reliabilities were still in the same level. As for within-modular degree, with the number of nodes changing from 90 to 1,024, its reliability gradually increased from moderate to excellent.

### Reliability of group-level modular-relevant analysis

**FIGURE 5:**
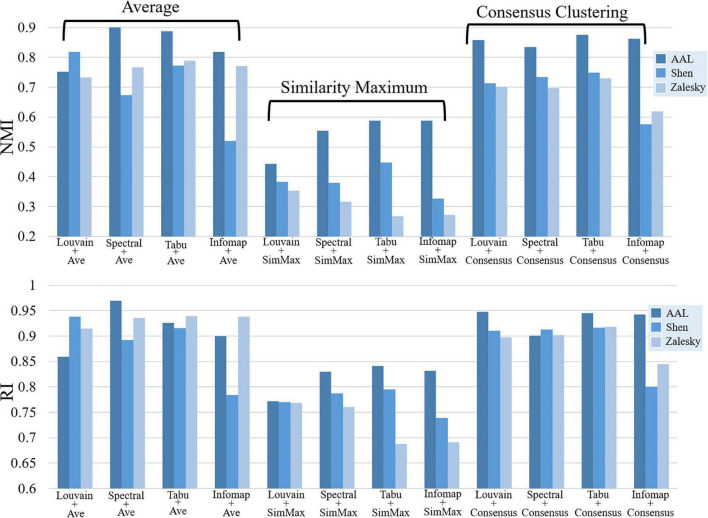
Repeatability of group-level module detection results estimated by normalized mutual information (NMI, **top**) and rand index (RI, **bottom**). Twelve module detection methods were applied for each brain atlas, including four average based methods, four similarity maximum based methods, and four consensus clustering based methods.

**FIGURE 6:**
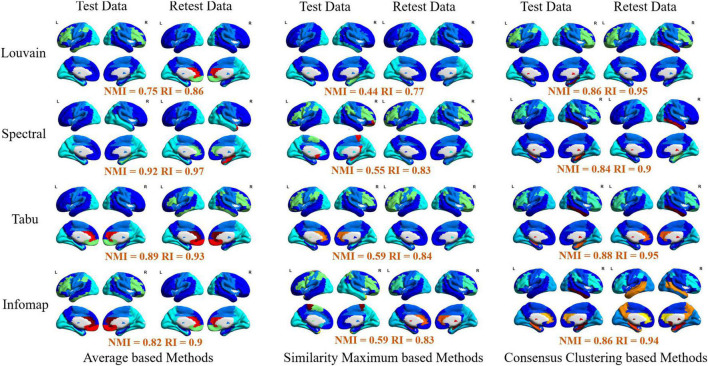
Visualization of group-level modular structure on automated anatomical labeling (AAL) atlas detected by 12 different module detection methods, including three average based methods (**left** two columns), three similarity maximum based method (**middle** two columns), and three consensus clustering based methods (**right** two columns). In each sub-figure, different colors represent different functional modules, and the similarity of module detection results between test and retest data estimated by normalized mutual information (NMI) and rand index (RI) were given below the figure.

**FIGURE 7:**
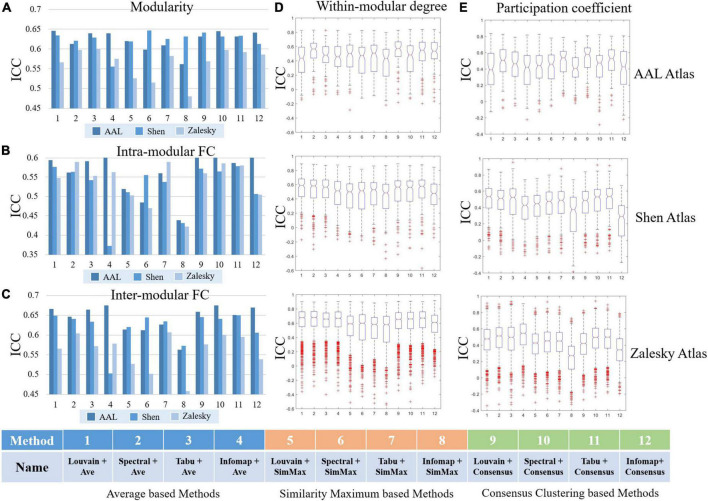
Test-retest reliabilities of group-level modular-relevant network metrics estimated by intra-class correlation (ICC), including **(A)** modularity, **(B)** intra-modular functional connectivity (FC), **(C)** inter-modular FC, **(D)** within-modular degree, and **(E)** participation coefficient.

In this section, we systematically evaluated TRT reliability of group-level modular-relevant analysis, including modular structure, modularity, intra- and inter-modular FCs, within-module degree and participation coefficient. Modular structure results were displayed in Figures 5, 6. Five network metric results were given in Figure 7 and Table 2.

**TABLE 2:** List of top four group-level module detection methods with the highest intra-class correlation coefficient (ICC) values on five network metrics estimated on three brain atlases.

Property	Atlas	ICC rank
Modularity	AAL	Louvain + Ave (good) > Spectral + Consensus (good) > Infomap + Consensus (good) > Tabu + Ave (good)
	Shen	Spectral + SimMax (good) > Louvain + Consensus (good) > Louvain + Ave (good) > Tabu + Consensus (good)
	Zalesky	Tabu + Ave (good) > Spectral + Ave (moderate) > Spectral + Consensus (moderate) > Tabu + Consensus (moderate)
Intra-modular FC	AAL	Infomap + Consensus (good) > Spectral + Consensus (good) > Infomap + Ave (good) > Louvain + Consensus (good)
	Shen	Tabu + Consensus (moderate) > Louvain + Ave (moderate) > Louvain + Consensus (moderate) > Infomap + Consensus (moderate)
	Zalesky	Tabu + SimMax (moderate) > Spectral + Ave (moderate) > Spectral + Consensus (moderate) > Tabu + Consensus (moderate)
Inter-modular FC	AAL	Infomap + Ave (good) > Spectral + Consensus (good) > Infomap + Consensus (good) > Tabu + Ave (good)
	Shen	Tabu + Consensus (good) > Louvain + Ave (good) > Louvain + Consensus (good) > Spectral + SimMax (good)
	Zalesky	Tabu + SimMax (good) > Spectral + Ave (good) > Spectral + Consensus (good) > Tabu + Consensus (moderate)
Within-module degree	AAL	Louvain + Consensus (moderate) > Spectral + Ave (moderate) > Tabu + Consensus (moderate) > Infomap + Consensus (moderate)
	Shen	Louvain + Ave (moderate) > Spectral + Ave (moderate) > Tabu + Consensus (moderate) > Louvain + Consensus (moderate)
	Zalesky	Louvain + Ave (good) > Spectral + Tabu (good) > Tabu + Consensus (good) > Infomap + Ave (good)
Participation coefficient	AAL	Louvain + Consensus (good) > Tabu + SimMax (moderate) > Tabu + Consensus (moderate) > Spectral + Ave (moderate)
	Shen	Louvain + Ave (moderate) > Spectral + Consensus (moderate) > Tabu + Consensus (moderate) > Tabu + Ave (moderate)
	Zalesky	Infomap + Ave (moderate) > Spectral + Ave (moderate) > Spectral + Consensus (moderate) > Tabu + Ave (moderate)

Poor reliability (0 < ICC < 0.2), fair reliability (0.2 < ICC < 0.4), moderate reliability (0.4 < ICC < 0.6), good (0.6 < ICC < 0.8), and excellent reliability (ICC > 0.8).

#### Modular structure

Figure 5 displayed the similarity of group-level modular structure between test and retest data obtained with 12 different module detection methods, including four average based methods, four similarity maximum based methods, and four consensus clustering based methods. In Figure 5, an obvious finding is that, both NMI and RI values derived from similarity maximum based methods were much lower than those from average based and consensus clustering based methods. It indicates that, in group-level module detection, the methods considering all subjects can yield more robust modular structure than those by selecting a representative subject. Further comparisons among four average based methods, we found the inconsistent result with individual-level analysis, i.e., none of the module searching approach displayed better performance than others in terms of repeatability of modular structure. The similar finding was also found among four consensus clustering based methods. All these results suggest that the repeatability of group-level network partition were sensitive to group-wise modular structure construction framework rather than module searching strategy.

Additionally, the effect of brain atlas was not exactly same as that found in individual level. In subject level, the modular structure detected on Shen atlas displayed the lowest repeatability among three brain atlases in most of experiments (as shown in Figure 2). However, in group-level analysis, the similar results were only found on three average based methods, i.e., Spectral + Ave, Tabu + Ave, and Infomap + Ave. For the other two frameworks, the repeatability of the resultant network partitions was decreased with the increase of network size.

Finally, for each atlas, we also projected 12 group-level network partition results obtained from different module detection algorithms on brain surface for the further comparison. The visualizations on AAL, Shen and Zalesky atlases were, respectively, given in Figure 6 and Supplementary Figures 2, 3. Similar to individual-level results, four functional subsystems could be also successfully captured by most of group-level module detection methods, i.e., VN, SMN, DMN, and FPN. Taking network partitions from Louvain algorithm as example, we further compared differences of spatial distribution of functional subsystems between different group-wise module construction schemes and different brain atlases. The network partition map generated by Yeo et al. (2014) were adopted as ground truth. In Figure 6, we observed two results. *First*, average and consensus clustering based methods yield the similar network partition results. *Second*, functional modules generated on Shen atlas were closer to the real spatial distributions than the other two atlases. For example, the results on AAL and Zalesky atlases assigned superior frontal areas to DMN, but they were not constituent brain areas of this system.

#### Modular-relevant network properties

We reported TRT reliability results of modularity, intra- and inter-modular FCs, within-modular degree, and participation coefficient estimated from group-level module detection in Figure 7. Besides, in all 15 experiments (5 network metrics ×3 brain atlases), we further sorted 12 group-level module detection methods based on ICC values and visualized the top four in Table 2 for the further comparisons.

As shown in Figure 7 and Table 2, the reliabilities of five group-level modular-relevant network metrics were ranged from moderate to good with modularity yielded the highest ICC values. These results suggest that, the estimation of network properties from group-level module detection in brain functional networks were reliable.

We further compared ICC values of 12 group-level module detection algorithms in each experiment, and found that reliability was relevant to group-wise module construction framework rather than module searching approach. Specifically, as shown in Table 2, among all listed module detection algorithms, there are 31 consensus clustering based methods, 24 average based methods, and 5 similarity maximum based methods, indicating the superiority of consensus clustering framework in terms of reliability assessment. However, the further comparison between four module searching approaches within each group-wise scheme did not find the significant difference between each other. Taking the consensus clustering framework as example, the proportions for Louvain algorithm, spectral method, Tabu method and Infomap algorithm were 7/31, 8/31, 11/31, and 5/31, respectively, and none of the method displayed significantly better performance than the others.

Regarding to the brain atlas, the similar results were found with individual-level analysis, that is, the reliability of most network properties derived from group-level modular detection was not significantly changed with the increase of network size. The only network metric with dramatic change was inter-modular FC, whose ICC values were slightly decreased with the increase of network size.

## Discussion

The main purpose of this study was to systematically investigate the reliability of modular-relevant analysis in brain functional network from rs-fMRI data, including modular structure and popular module-derived network metrics. We were also interested in evaluating how module detection algorithm and node definition approach affect the reliability. Through a series of experiments, we drew the following conclusion. *First*, five modular-relevant network metrics were reliable and reproducible with TRT reliability ranging from moderate to good. *Second*, the reliability of network metrics could be affected by module detection algorithm but there was difference between individual- and group-level analyses. Individual-level reliabilities were sensitive to module searching strategy, whereas group-level reliabilities depended heavily on group-wise modular structure construction framework. *Third*, node definition approach was another potential affecting factor for reliability of modular-relevant analysis, and both the generation approach and parcellation granularity of brain atlas could impact ICC values.

To our best knowledge, there is no such comprehensive reliability study before specific to mesoscale module-related network measuring approaches from rs-fMRI data. Although many studies have investigated reliabilities of FCs and network properties at macroscale and microscale (Shehzad et al., 2009; Anderson et al., 2011; Shou et al., 2013; Mueller et al., 2015; Shirer et al., 2015; Laumann et al., 2017; Tomasi et al., 2017), they cannot guarantee the repeatability of module detection results and their derived network analysis approaches. This study filled this gap that focused on this important mesoscale graph theory tool to provide a potential guidance for the usage of module detection in brain functional networks in future studies.

### Reliability of modular-relevant network analysis

The results from a series of reliability evaluation experiments indicate five popular network measurements from functional modules are reliable and reproducible. This finding not only fills the gap of mesoscale reliability assessment of brain functional networks, but also provides an evidence for the potential application of module detection in future clinical trials. Of the five network metrics, we noted that modularity yields the highest ICC values. This is because it is calculated by directly comparing the modular structure of the given network and stochastic model (Sporns and Betzel, 2016), thus avoiding the secondary introduction of unreliable FCs to reduce reliability values. In addition, the present work reported good-to-excellent reliability in modularity, which were much higher than the previous studies (Braun et al., 2012; Du et al., 2015). This discrepancy may due to different imaging acquisition parameters and network analysis approaches. Specifically, compared with Braun et al. (2012), the higher reliability in our work to some extend is benefited from better rs-fMRI data acquisition parameters, including subject’s eye open and much more frames per subject, etc. A recent FC reliability literature review in support of this view. It using the meta-analysis method concluded that studies with rs-fMRI collected in subject awake, eye open and more within-subject data tend to generate more reliable FCs (Noble et al., 2019), and thus more reproducible network properties. As for Du et al. (2015), the main reason for its poorer reliability is due to the large voxel-wise brain functional network intensifies the non-determinacy of module detection algorithm, making it difficult to obtain a stable and accurate modular partition. This gives us a hint of the care should be taken of voxel-wise analysis when using graph theory tools of module detection in brain functional networks.

Regarding regional-level module-derived network metrics, we found both within-modular degree and participation coefficient from brain functional networks were less reliable than those from structural networks. Comparing to moderate-to-good reliability from rs-fMRI in present work, a recent reliability assessment in structural brain network from diffusion tensor imaging (DTI) using the similar approach reported good TRT reliability in within-modular degree and excellent reliability in participation coefficient on AAL atlas (Dimitriadis et al., 2021). This finding proves again that structural connectivity (SC) from DTI is more robust than FC from rs-fMRI as previous studies announced good reliability in SC of averaged ICC = 0.62 (Buchanan et al., 2014) versus poor reliability in FC of averaged ICC = 0.29 (Noble et al., 2019).

### Effects of module detection algorithm

We found that module detection algorithm has a significant influence on the reliabilities of modular-relevant network measurements. This is not surprising because the repeatability of network partition results on test and retest data heavily determines the reliability of network attributes derived from them. An interesting finding is that module detection method affects individual- and group-level analyses through different manners. Individual-level results reported Louvain algorithm performed better than the other methods in almost all experiments (as shown in Figure 4), suggesting module searching strategy has a great impact on reliability of network metrics. However, group-level experiments did not find the superiority of any module discovery method, but the group-wise representative module determination framework significantly affect the ICC values (e.g., average and consensus clustering based frameworks perform much better than the similarity maximum as shown in Figure 7). This difference is due to the fact that individual-level module detection algorithm needs to ensure the partition quality of each tested brain functional network; while group-level detection aims to obtain consistent partition results across the population group, which has a relatively low requirement for the partition accuracy of each single network. The above findings can provide a guidance for the selection of module detection algorithms in the subsequent brain functional network research.

Of four individual-level module detection algorithms, Louvain algorithm obtained the best results in almost all brain networks ranging from small to large networks. This is due to its hierarchical module searching strategy, which not only makes it easier to approach the optimal solution in NP-hard problems, but also suitable for large-scale networks (Blondel et al., 2008). In contrary, Tabu method can only work well on small brain network limited by its exhaustive searching approach, and spectral method is not prominent in all networks. A more interesting finding is that, in terms of reliability, Infomap method is not suitable for module detection in brain functional networks. We speculate the main reason is that the random walk used in this method is highly dependent on the reliability of edges in network, but FCs are usually unstable due to imaging noise (Birn, 2012; Murphy et al., 2013). One possible solution is to improve the stability of the function connection or use a binary network. In group-level analysis, the average and consensus clustering based module detection algorithms yielded comparable performance in reliability assessment, which is much better than those using similarity maximum as group-wise module determination framework. This conclusion is in line with our expectation because the similarity maximum framework is to select network partition from one subject to represent group-level modular structure, which is difficult to characterize the module characteristics of all the tested brain functional networks.

### Effects of node definition approach

While the impact of node definition approach on the topological organization of brain functional networks has been widely acknowledged (Wang et al., 2009; Fornito et al., 2010; Zalesky et al., 2010; Power et al., 2011), the question of how parcellation scheme influences the reliability is still unclear. Most studies assessed the reliability of network metrics using common AAL atlas (Schwarz and McGonigle, 2011; Braun et al., 2012; Chou et al., 2012; Guo et al., 2012; Liang et al., 2012; Fiecas et al., 2013; Jin et al., 2018), and only few studies have focused on this feature (Wang et al., 2011; Cao et al., 2014). These studies compared the performance between structural [AAL atlas and Harvard-Oxford atlas (Kennedy et al., 1998)] and functional atlas [F-DOS (Dosenbach et al., 2010) and funROI (Power et al., 2011)], and concluded that the reliability of network metrics is certainly influenced by the chosen brain atlas, but it is difficult to define which parcellation scheme yields the highest reliability. Similar conclusion were reached in our work that the robustness of modular-relevant analysis was modulated by strategies of network node definition, and none of brain atlas outperformed the others in all cases. For example, in the individual level, Shen atlas displayed the lowest repeatability in network partition results (shown in Figure 2), whereas the group-level module detection found Zalesky atlas performed worst. The discrepancy between individual and group levels may due to that the reliability of modular-relevant analysis can be influenced by both parcellation granularity of the brain atlas and its generation approach. On one hand, it is well known that increasing network size would worsen the uncertainty of module detection results, thus the brain atlas with finer parcellation generally display lower repeatability than coarser parcellation. Therefore, in group-level modular analysis, the Zalesky atlas with the largest number of nodes exhibited the lowest reliability. On the other hand, in terms of atlas generation, ROIs from AAL, Shen and Zalesky atlases were, respectively, obtained based on anatomical features of sulcal pattern (Tzourio-Mazoyer et al., 2002), brain region’s functional connectivity, and geometric information. A previous study found that the geometric parcellation could yield more reliable FCs than the data-driven FC-based parcellation scheme (Zeng et al., 2019). This may be the reason of the poorer performance of Shen atlas than Zalesky atlas in individual-level analysis. Recent studies have also attempted to determine the best brain parcellation in terms of quality and reliability but failed to figure out a clear winner (Arslan et al., 2018), which needs to be further investigated in future studies.

### Limitations and technique considerations

Our study has a few of limitations. *Firstly*, we only explored the influence of module detection algorithm and node definition approach on reliability of modular-relevant network metrics, without going through imaging acquisition and preprocessing procedures. Previous studies have identified differences in acquisition and processing procedures also impact test-retest reliability (Zuo et al., 2013; Aurich et al., 2015; Varikuti et al., 2017), e.g., temporal resolution of the fMRI data (Birn et al., 2014; Shah et al., 2014; Zuo and Xing, 2014), global signal regression (Shirer et al., 2015; Varikuti et al., 2017), and motion correction (Zuo and Xing, 2014), etc. Hence, the reliability results in this work are specific to the acquisition protocol of HCP dataset and specific imaging preprocessing. *Secondly*, the reproducibility of brain functional networks can be affected by FC estimation methods (Liang et al., 2012) and network sparsity strategies (Cao et al., 2014). These variables should also be considered in functional brain networks following our data-driven analysis. However, our work only examined two factors (i.e., module detection algorithm and node definition approach) which can impact the repeatability of network partitions directly. *Lastly*, using rs-fMRI, we examined the reliability of modular-relevant analysis in functional brain networks. Previous studies have performed similar analyses of structural brain networks using DTI data (Dimitriadis et al., 2021). A systematic reliability evaluation using multimodal data from the same population is warranted to gain a deeper understanding of the structural and functional architecture of the human brain.

## Conclusion

In this study, we systematically investigated test-retest reliability of diverse modular-relevant analyses in brain functional networks as well as how they are affected by module detection algorithm and node definition approach. The results showed that five popular modular-derived network metrics could provide robust results with modularity achieving the highest reliability. The extensive analysis identified different module detection methods and brain atlases yield different reliability in network metrics. Our analysis indicate that much attention should be paid to the choice of module detection algorithms and node definition approaches in order to extract reliable results at the mesoscale of brain networks.

## Data availability statement

The original contributions presented in this study are included in the article/Supplementary material, further inquiries can be directed to the corresponding author.

## Ethics statement

The studies involving human participants were reviewed and approved by Human Connectome Project, WU-Minn Consortium. The patients/participants provided their written informed consent to participate in this study.

## Author contributions

XW and MY developed, implemented, and evaluated the algorithms, wrote, and revised the manuscript. LH and DZ reviewed and supervised the research. All authors contributed to the article and approved the submitted version.

## Footnotes

1
https://www.humanconnectome.org/study/hcp-young-adult
