# Supplementary material for: Test-retest reliability of modular-relevant analysis in brain functional network
Source: Front Neurosci. 2022 Dec 7;16:1000863. doi: 10.3389/fnins.2022.1000863 (PMC9770801; doi:10.3389/fnins.2022.1000863)
Supplement: Supplementary file 1 [file Image_1.PDF]

Supplementary Materials

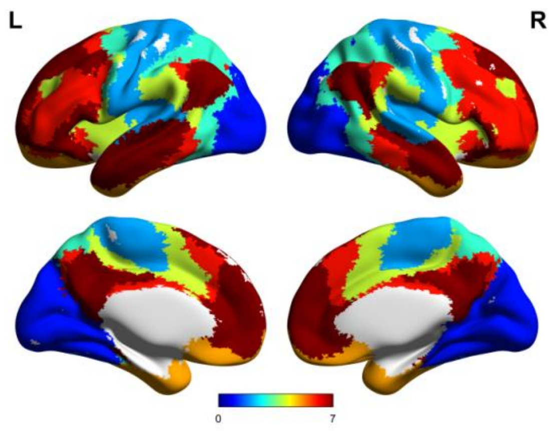

Supplementary Figure S1 Functional Partition map of the human brain obtained by Yeo and his colleagues.

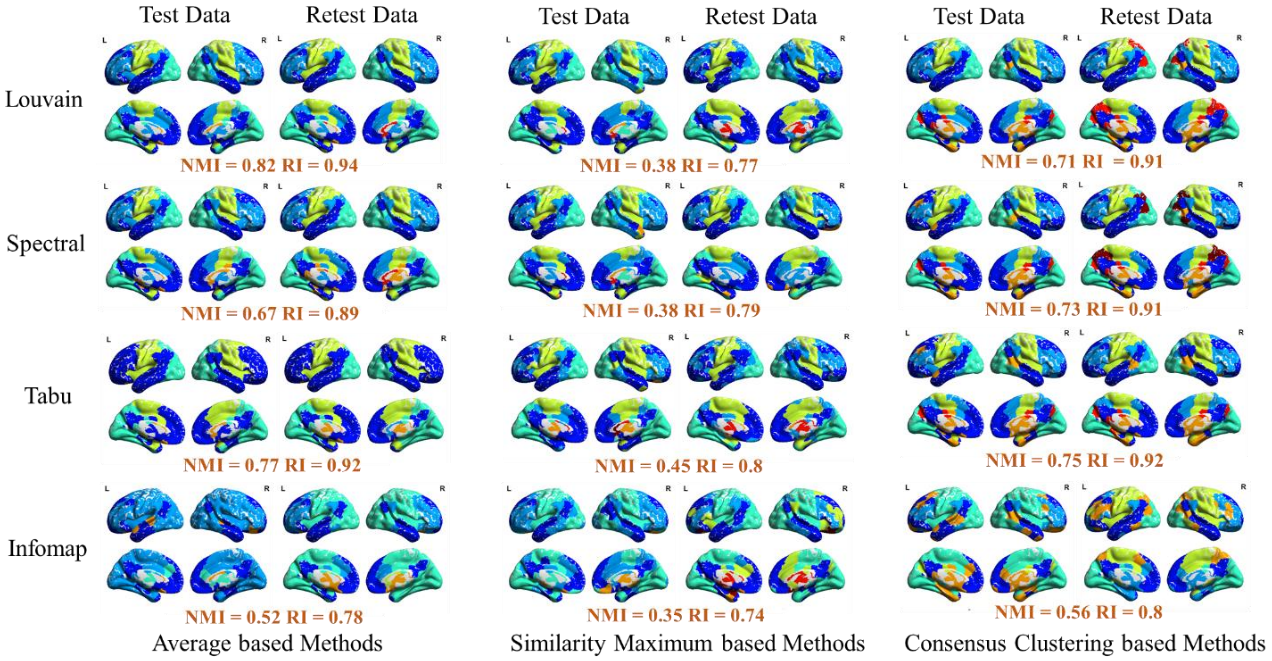

Supplementary Figure S2 Visualization of group-level modular structure on Shen atlas detected by twelve different module detection methods, including three average based methods (*left* two columns), three similarity maximum based method (*middle* two columns), and three consensus clustering based methods (*right* two columns). In each sub-figure, different colors represent different functional modules, and the similarity of module detection results between test and retest data estimated by normalized mutual information (NMI) and rand index (RI) were given below the figure.

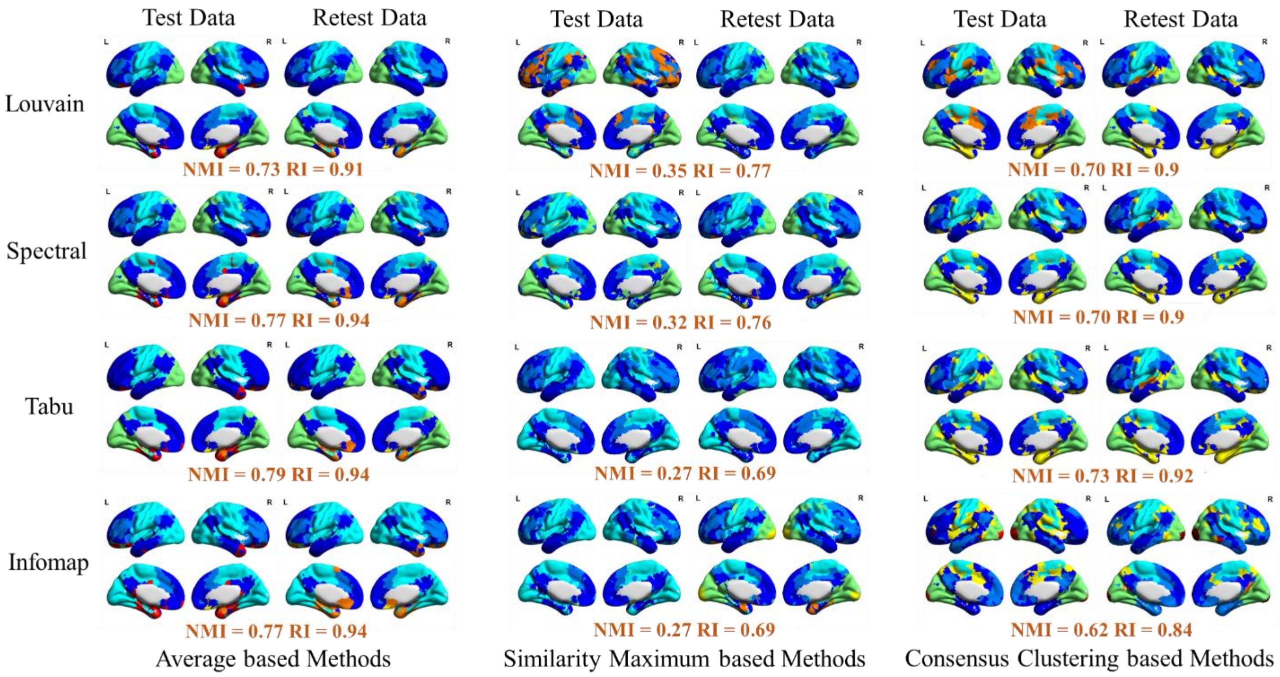

Supplementary Figure S3 Visualization of group-level modular structure on Zalesky atlas detected by twelve different module detection methods, including three average based methods (*left* two columns), three similarity maximum based method (*middle* two columns), and three consensus clustering based methods (*right* two columns). In each sub-figure, different colors represent different functional modules, and the similarity of module detection results between test and retest data estimated by normalized mutual information (NMI) and rand index (RI) were given below the figure.
